# Guidelines for Designing Surface Ion Traps Using the Boundary Element Method

**DOI:** 10.3390/s16050616

**Published:** 2016-04-28

**Authors:** Seokjun Hong, Minjae Lee, Hongjin Cheon, Taehyun Kim, Dong-il “Dan” Cho

**Affiliations:** 1 ISRC/ASRI, Department of Electrical and Computer Engineering, Seoul National University, Seoul 151-744, Korea; sjhong84@snu.ac.kr (S.H.); mjlee88@snu.ac.kr (M.L.); hjcheon@snu.ac.kr (H.C.); 2 Quantum Tech. Lab., SK Telecom, Seongnam-si, Gyeonggi-do 463-784, Korea

**Keywords:** surface ion trap, electrode dimensions, design optimization, boundary element method, microfabrication

## Abstract

Ion traps can provide both physical implementation of quantum information processing and direct observation of quantum systems. Recently, surface ion traps have been developed using microfabrication technologies and are considered to be a promising platform for scalable quantum devices. This paper presents detailed guidelines for designing the electrodes of surface ion traps. First, we define and explain the key specifications including trap depth, *q*-parameter, secular frequency, and ion height. Then, we present a numerical-simulation-based design procedure, which involves determining the basic assumptions, determining the shape and size of the chip, designing the dimensions of the radio frequency (RF) electrode, and analyzing the direct current (DC) control voltages. As an example of this design procedure, we present a case study with tutorial-like explanations. The proposed design procedure can provide a practical guideline for designing the electrodes of surface ion traps.

## 1. Introduction

An ion trap is a device to trap charged particles in space using an electric or electromagnetic field. In particular, if atomic ions are trapped in an ultra-high vacuum environment, it can provide ideal isolation from the surroundings and allows individual manipulations of the trapped ions. These manipulations can be achieved using either a focused laser with beam-steering capability [1] or an optimized design of the microwave near field at trapped-ion location [2]. Because of these features, the ion trap method is considered a promising candidate for the physical implementation of quantum information processing [3] and a precise measurement system for fundamental physics applications such as the optical clock and mass spectroscopy [4,5,6].

**Figure 1:**
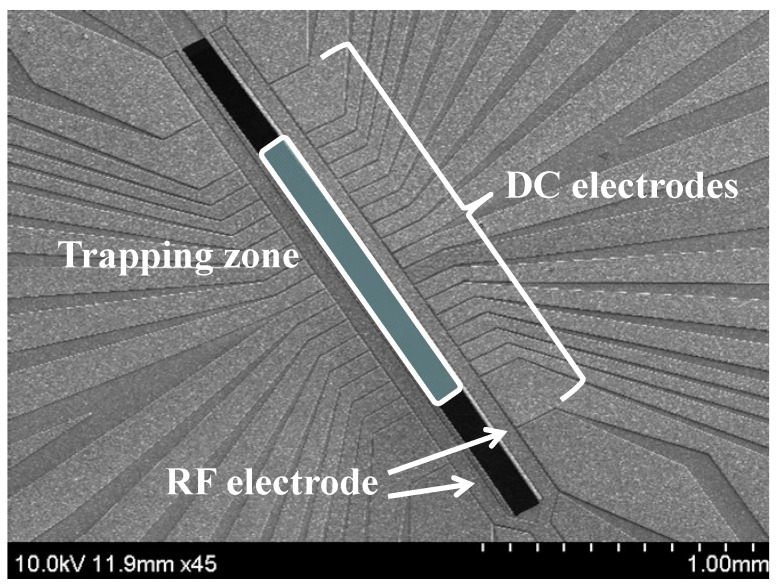
Scanning electron micrograph image of a surface ion trap fabricated by our group [11]. (The design layout of the ion trap uses the trap chip of Sandia National Laboratory [12] as the benchmark.)

Earlier ion traps were constructed using conventional machining and manual assembly. Following the proposal for a multi-zone ion trap with a scalable architecture for complex quantum information processing [7], microfabrication technologies have become methods of choice for realizing various ion traps [8]. In microfabrication technologies, the electrodes of conventional ion traps, which have a three-dimensional quadrupole configuration, are projected onto a plane. This allows for a lithography-based fabrication method that can significantly reduce alignment errors during manual assembly and provide scalability [9]. Microfabricated ion traps whose electrodes are laid on a plane are known as “surface ion traps” or “planar ion traps” (Figure 1). Currently, surface ion traps are widely used as platforms for quantum information processing [10,11,12]. In addition, the surface ion traps have potentiality in the realization of portable optical clocks, because the ion-trap system can be miniaturized by adopting the surface ion traps [13].

With respect to the electrode design for surface ion traps, several previous studies present analytical models [14,15,16,17,18]. However, these models cannot be simply applied in practical designs due to the complex structures of surface ion-trap chips. There also have been many studies on designing electrode dimensions using numerical simulations. Pearson *et al.* extracted specifications including trap depth and secular frequency through boundary element method (BEM) simulations [19]. Brownnutt *et al.* used a simulation method that uses the sum of the electrical potentials generated by individual electrodes [20]. Allcock *et al.* used BEM simulations to calculate direct current (DC) voltage sets for shaping DC potentials [21]. Nizamani and Hensinger presented a geometric factor in the electrode dimensions that provides the maximum trap depth when the ion height is determined [22]. Siverns *et al.* presented the relation between ion height and secular frequency in numerically simulated five-rail geometries [23]. Doret *et al.* proposed an iterative BEM method for designing radio frequency (RF) electrodes of a complex shape that compensates for the distortion of the RF pseudopotential near the backside loading slot [24]. This iterative BEM method can also be applied to design the RF electrodes of junction ion traps to decrease the amplitude of the pseudopotential barrier near the junction center [25,26,27]. In addition, a few Ph.D. dissertations have proposed more detailed electrode design methods for surface ion traps [28,29,30,31].

Table 1 summarizes the electrode dimensions and trap specifications of surface ion traps from recent publications [12,21,24,25,27,32,33,34]. As shown in the table, most papers addressing electrode design for surface ion traps do not provide any characteristics of surface ion traps. Although several research groups have used numerical simulations to design electrodes, they have provided only partial aspects of these simulations [35], and as yet, no comprehensive design procedure is available.

In this paper, we present a detailed design procedure for surface ion-trap chips. In Section 2, we provide the background of surface ion traps and the specification requirements. In Section 3, we describe our step-by-step design methodology for surface ion-trap chips using numerical simulations. In Section 4, we present a case study for our design methodology. We believe that the results of this study can provide an effective guideline for designing surface ion traps.

## 2. Background

### 2.1. Principle of Surface Ion Traps

**Figure 2:**
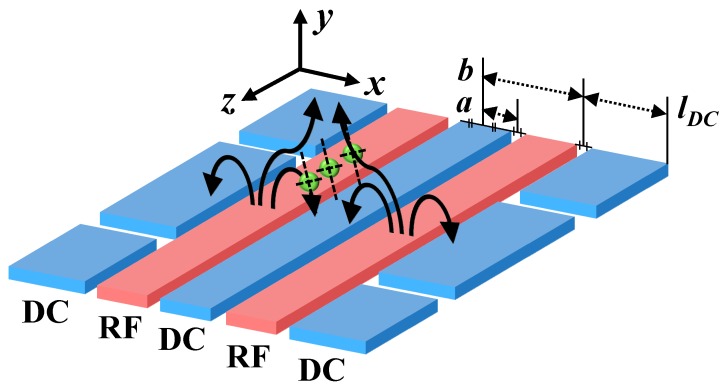
Schematic of a surface ion trap in a symmetric five-rail geometry. The red and blue rectangles indicate the RF and DC electrodes, respectively. The curved arrows denote the direction of the electric field when the RF voltage is positive. The dashed black lines indicate the principal axes of ion motions, which are tilted, for Doppler cooling with a single laser.

Figure 2 shows a schematic of a surface ion trap in a symmetric five-rail geometry [17]. By applying RF voltages to the two RF electrodes and maintaining the center and outer electrodes at RF ground, electric pseudopotentials are formed, which confine the trapped ions in radial directions. The outer electrodes are segmented to control the longitudinal electric potentials, which either confine the trapped ions or shuttle them along the axial direction. The center electrode can be divided into two separate electrodes. The separated center electrodes allow for tilting of the principal axes, which, in turn, allows Doppler cooling of the trapped ions with a single laser [21]. Note that the axis tilting can be achieved by applying asymmetric voltage on the center or outer electrodes.

### 2.2. Specifications of Surface Ion Traps

#### 2.2.1. Trap Depth

**Figure 3:**
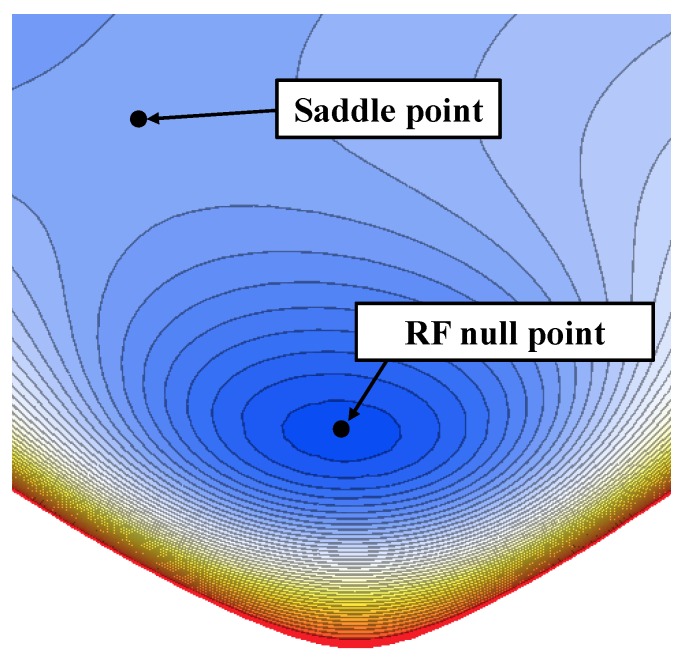
Contour plot of total potential generated by a surface ion trap indicating the RF null point and saddle point. This figure was obtained by boundary element method (BEM) simulations.

Trap depth is the difference in the total potential (sum of electric pseudopotential and DC potential) between the RF null and the saddle point, which is a stationary point but not a local extremum (Figure 3). Deeper trap depths suppress the loss of ions induced by background gas collisions and increase ion lifetime. Surface ion traps have an inherently shallower trap depth that is barely above the mean kinetic energy of the evaporated neutral atoms [17]. Therefore, maximization of trap depth is frequently considered as a design goal for a surface ion trap.

#### 2.2.2. *q*-Parameter

The classical radial motions of ions trapped in hyperbolic electrodes can be described by the standard Mathieu differential equation [36]:

(1) $$\frac{d^{2}i}{d\tau^{2}}+\left(a_{i}-2q_{i}\cos2\tau \right)\;i=0,\;\;\;i=x,y$$

where *τ*, *a_x_*, *a_y_*, *q_x_*, and *q_y_* are given below:

(2) $$\tau =\frac{\Omega t}{2},\;\;a_{x}=-a_{y}=\frac{4eU}{mr_{0}{}^{2}\Omega^{2}},\;\;q_{x}=-q_{y}=\frac{2eV}{mr_{0}{}^{2}\Omega^{2}}$$

and Ω, *t*, *e*, *m*, *r*_0_, *U*, and *V* indicate the RF voltage angular frequency, time, elementary charge, ion mass, ion–electrode distance, offset voltage, and RF voltage amplitude, respectively. In addition, the *q*-parameter is related to the secular frequencies and amplitudes of ion micromotion. The motion of the ion in the *x*-direction is described as follows [36]:

(3) $$\begin{array}{l} x=x_{0}\cos\left(\beta_{x}\frac{\Omega }{2}t\right)\left[1-\frac{q_{x}}{2}\cos\left(\Omega t\right)\right],\;\;\omega_{s,\;x}=\frac{\beta_{x}\Omega }{2}\\ \beta_{x}\approx \sqrt{a_{x}+\frac{q_{x}{}^{2}}{2}},\;\;q_{x}\approx 2\sqrt{2}\frac{\omega_{s,\;x}}{\Omega }\;\left(\text{when}\;a_{x}=0\right) \end{array}$$

where *x_0_* and *ω*_s,*x*_ indicate the oscillation amplitude of secular motion along the *x*-direction and the secular frequency along the *x*-direction, respectively. The ion motion in the *y*-direction can be described similarly. Moreover, these formulae are approximations. The ion motion comprises a secular motion at frequency *β_x_*Ω/2 ≪ Ω and a micromotion at frequency Ω. Generally, a higher secular frequency is preferred for the reason described in Section 2.2.3, while a smaller *q*-parameter is preferred to minimize micromotion amplitude. However, they cannot be achieved simultaneously as evident from Equation (3), unless RF angular frequency is increased. However, RF angular frequency is also limited by the maximum allowed RF voltage and the minimum desired trap depth as discussed later. Typically, the stable region usually used for hyperbolic electrode traps includes *a* and *q*, where *q* < 0.7 and |*a*/*q*^2^| < 0.5 [16]. However, the stable region of *a* and *q* for a surface ion trap varies with the tilted angle of the principal axes, which is dependent on the geometries of the DC electrodes and the arrangement of the DC voltage [37]. Because of the calculation complexity of the analytic derivation, an appropriate *q*-parameter value between 0.1 and 0.3 is generally considered as a starting point for design, and an optimal *q*-parameter can be determined as the experiment proceeds within stable ranges. This *q*-parameter range is consistent with other examples listed in Table 1.

#### 2.2.3. Secular Frequency

Secular frequency can be derived from the Mathieu parameters, as shown in Equation (3), and can be expressed as follows:

(4) $$\omega_{s,\;i}=\frac{e}{m}\frac{d^{2}\phi }{di^{2}},\;\;i=x,y,z$$

where *φ* is the total electric potential, which is the sum of the electric pseudopotential and static potentials. A higher secular frequency is preferred because it allows for tighter confinement, faster ion transportation [38], better cooling and less sensitivity to external noise [39]. Higher secular frequencies are also preferable for multi-qubit entangling gate operations utilizing motional modes [40].

Radial secular frequencies are strongly influenced by the electrode dimensions and are considered in the design of RF electrodes. The axial secular frequency is determined by adjusting both the DC voltages and the electrode geometry. When the peak voltage on the DC electrodes are limited by digital-to-analog converters, the maximum electric potential generated by a given electrode geometry can also be limited. Thus, occasionally, the designed electrode dimensions do not provide sufficiently high axial secular frequencies. Therefore, the effects of the DC voltage at the RF null point must be investigated after designing the RF electrodes. We explain this further in Section 3.4.

#### 2.2.4. Ion Height

As shown in Figure 2, the RF and DC electrodes of the surface ion trap are laid on the same plane and the RF null point is placed above the plane. The ion height is the vertical distance between the RF null point and the plane. Higher ion heights correspond to slower motional heating rates of the trapped ions [14]. However, a higher height also corresponds to a weaker trapping pseudopotential at the RF null point.

**Figure 4:**
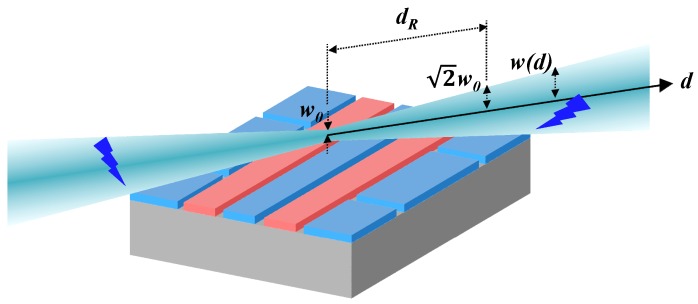
Schematic of a Gaussian beam injected parallel to the surface ion trap. The blue lightning symbols indicate the scattering of the Gaussian beam by the surface ion-trap chip at the chip edges.

The beam size of the lasers must also be considered when selecting the ion height. We describe the radius of a Gaussian beam at a distance *d* from the waist, *w*(*d*), as follows:

(5) $$w\left(d\right)=w_{0}\sqrt{1+{\left(\frac{d}{d_{R}}\right)}^{2}},\;\;d_{R}=\frac{\pi w_{0}{}^{2}}{\lambda }$$

where *w*_0_ is the beam waist, which is the radial size of the beam at its narrowest point, and *λ* is the wavelength [41]. Equation (5) indicates that a thinner beam waist can accompany a larger beam radius at the edge of the surface ion-trap chip. Figure 4 shows a schematic of a Gaussian beam injected parallel to the surface ion trap. An increase in the beam size induces beam clipping by the chip body, which can increase the laser scattering caused by the diffraction of the clipped laser. By setting the Rayleigh length of Gaussian beam (*d_R_*) equal to half the traverse distance of the beam propagation above the chip as shown in Figure 4, the disturbance to beam propagation by the chip can be minimized. In addition, the ratio of ion height over beam size at the edge of the chip allows us to estimate the amount of potential laser scattering due to laser clipping.

### 2.3. Analytic Solutions

Based on a five-rail geometry, which assumes that the electrode surfaces are infinite and that there are no gaps between electrodes, the specifications of the surface ion trap described in the previous subsections are analytically solved as follows [15]:

(6) $$\begin{array}{l} \text{trap}\;\text{depth}=\frac{eV^{2}}{m\Omega^{2}}\frac{{\left(a-b\right)}^{2}}{\pi^{2}{\left(a+b\right)}^{2}\left(a^{2}+6ab+b^{2}+4\sqrt{ab{\left(a+b\right)}^{2}}\right)}\\ \text{q-parameter}=\frac{eV}{m\Omega^{2}}\frac{8(b-a)}{\pi \sqrt{ab}{\left(a+b\right)}^{2}}\\ \text{ion}\;\text{height}=\sqrt{ab} \end{array}$$

where *a* and *b* are indicated in Figure 2. The secular frequency can be calculated by combining Equations (3) and (6). However, these analytic solutions are not valid for the complex structures of actual surface ion traps, including the loading slot, finite width of outer electrodes and segmentation, and multi-layered electrodes, because certain assumptions do not hold for these complex structures. Nonetheless, these analytic solutions are very useful for making a rough first design of a surface ion trap before applying the numerical optimization procedures outlined in the following sections.

### 2.4. BEM Simulations

The application of advanced microfabrication technologies has allowed the development of complex ion trap structures such as loading slots, junctions, and even three-dimensional quadrupole structures [24,25,26,27,42]. Complex ion trap structures are difficult to be analytically modeled; therefore, simulations and numerical analyses need to be used. BEMs have been shown to provide more precise and faster simulations of a surface ion trap than finite element methods [21,28]. In this paper, we use the BEM-based software Charged Particle Optics (CPO Ltd., Manchester, UK). We can simulate the electrical potential and electric field induced by each electrode by applying 1 V to the electrode being measured while grounding the other electrodes. Then, the results are scaled and summed in order to calculate the total electric potential. The pseudopotential *φ*_pp_(**r**) generated by the trap RF is defined by:

(7) $$\phi_{\mathrm{pp}}\left(r\right)=\frac{e^{2}{\left|E\left(r\right)\right|}^{2}}{4m\Omega^{2}}$$

where **r** represents the position vector and *E*(**r**) represents the RF electric field amplitude generated by applying voltage *V* on RF electrodes [14]. From the estimated total electric potential, we can calculate the specifications explained in Section 2.2, including trap depth, *q*-parameter, secular frequencies, and ion height.

**Figure 5:**
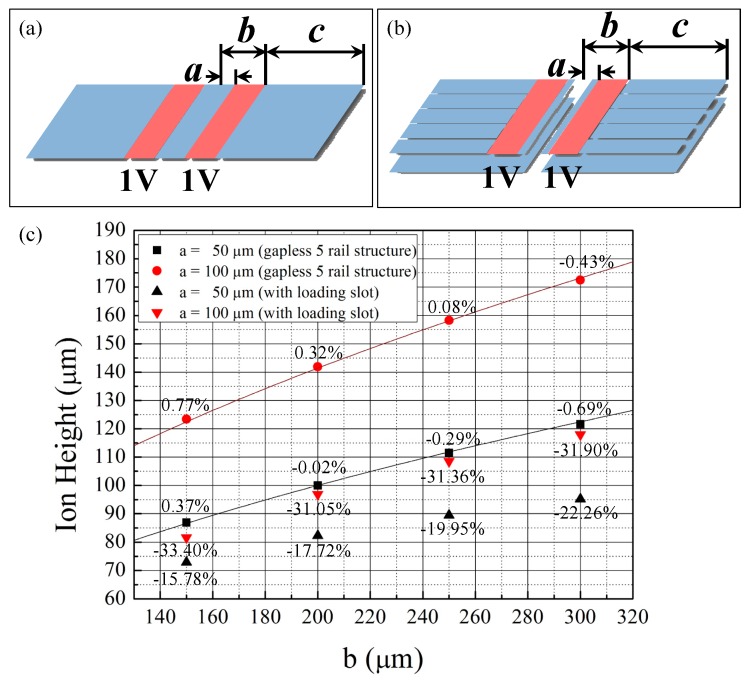
Simulation results of the ion heights for simple and complex electrode geometries. (**a**) a simple electrode geometry with 1-μm electrode gaps; (**b**) a complex electrode configuration with inner DC rails, a loading slot, and ground planes. The width of the inner DC rails is 20 μm regardless of a. The widths of the loading slot for the case of a = 50 μm and 100 μm are 56 μm and 156 μm, respectively; (**c**) simulation results of the ion heights. The black and red lines represent the analytic solution of the ion heights for the ideal five-rail geometries when *a* = 50 μm and 100 μm, respectively. The numbers near the bullets represent the errors between the analytic solution for the ideal five-rail geometries and the simulation results with the same *a* and *b* values.

To determine the validity of the BEM tool, we simulate the electric potential generated by a simple electrode structure with 1-μm electrode gaps, as shown in the upper-left inset of Figure 5. This figure shows the analytic solution from Equation (6) and the simulated ion heights. If we use a uniform segment size of 3000 μm^2^ for both the RF and DC electrodes, the maximum error between the analytic and simulated values is 1.62%. When the segment size is adjusted to 333−357 μm^2^ for the RF electrodes and 6800−7400 μm^2^ for the DC electrodes, the maximum error is reduced to 0.77%, as shown in Figure 5. The computational requirements for the two cases are 348 MB memory for 12 min and 611 MB memory for 50 min, respectively. We perform the simulations using an i-7 4790 processor (Intel Corporation, Santa Clara, CA, USA). The adaptive mesh function of the BEM tool can also be used. In this case, the maximum error is 0.83% and the computational requirements are 636 MB memory for 60 min. As described above, by adjusting the segment size both manually and automatically, the differences between the analytic solutions and the simulation results can be reduced for cases where *a* = 50 and 100 μm and *b* = 150, 200, 250, and 300 μm. Therefore, the simulation results using the BEM tool seem valid for estimating the electric potentials generated by trap-chip electrodes. However, when *a* is fixed at 50 or 100 μm and *b* becomes very small or large, the differences between the analytic solutions and simulation results seem to increase. This is because the widths of the simulation regions are fixed at 1 mm in each simulation set, and that of the outer ground plane is decreased as *b* increases. For such cases, the simulation regions must also be adjusted to provide an outer ground plane of appropriate size. The simulation results in Figure 5 show that the error between the analytic solutions and simulation results can be minimized when *c* ≈ 2*b*, where c is the width of the outer ground plane (or the length of the DC electrodes of segmented traps).

Next, we simulate the electric potential generated by a more complex electrode configuration shown in the upper-right inset in Figure 5, to determine the need for numerical simulations. The electrode structure includes two inner DC rails, an ion-loading slot between the rails, and ground planes. Moreover, the ground planes are placed 14 µm below the top electrode. The width of the inner DC rails is 20 μm regardless of *a*. The widths of the loading slot for the case of *a* = 50 μm and 100 μm are 56 μm and 156 μm, respectively. In this case, the differences between the analytic solutions and the BEM-simulated results are significant, as shown by the black and red triangular marks in Figure 5. Thus, using the BEM method is essential when designing surface ion traps with complex configurations.

## 3. Design Methodology for Surface Ion-Trap Chips

The design procedure in this paper follows four steps: setting the basic assumptions, determining the shape and size of the chip, designing the dimensions of the RF electrodes, and investigating the effects of the DC voltages at the ion position.

### 3.1. Basic Assumptions

First, we must establish some basic assumptions that are independent of the design layout. The ion species used in the ion-trap experiments determines the wavelengths of the lasers to be used. The ion mass is needed to calculate the trap specifications. The type of the ceramic chip package limits the maximum chip size and number of electrodes. In addition, to accurately simulate the electric potential, the thicknesses of the conducting films and the insulator materials must be defined.

Next, we must determine the constraints of the design parameters including *a*, *b*, *V*, and Ω. The lengths *a* and *b* in Figure 2 determine the distance between the two RF electrodes and the distance of the outer DC electrodes from the ion position, respectively. Between the RF electrodes, we can position a loading slot and the center DC electrodes. Thus, we determine *a* by the widths of the loading slot and the center DC electrodes. The width of the loading slot can be designed with respect to the configuration of the components that supply neutral atoms, and are typically tens of micrometers. The role of the center DC electrodes is to generate an electric potential that can compensate for the asymmetry of the electric potential generated by the outer DC electrodes. If *b* increases, the electric field from the outer DC electrodes is reduced, which reduces the width of the center DC electrodes to keep the ion height constant. Essentially, the constraints for *a* and *b* are related. The optimal constraint dimensions for *a* and *b* cannot be determined in this step. Instead, the constraint dimensions are initially established by referring to previous work and must be optimized by repetitively designing the RF electrodes and DC voltage set. The maximum value of *V* is restricted to prohibit electric breakdown between the electrodes and the ground plane. The RF frequency Ω can be easily adjusted without many constraints. Both *V* and Ω affect the trap depth, *q*-parameter, and radial secular frequencies. The trap depth, radial secular frequencies, and *q*-parameter are proportional to *V*^2^/Ω^2^, *V*/Ω, and *V*/Ω^2^, respectively. Thus, *V* is assumed to be the maximum value and Ω is adjusted to achieve a low *q*-parameter and high trap depth and radial secular frequencies. We cannot calculate the RF breakdown voltage because the Paschen curve may not be applicable in an ultra-high vacuum environment [43]. However, it has been reported that an alternating current (AC) field of ~30 V µm^−1^ can induce electric breakdown between sharp electrodes in vacuum [44,45]. Because an electrode gap of ~10 µm is generally used in surface ion traps to prevent electric breakdown between the electrodes and considering the microfabrication constraints, the RF amplitude is restricted to ~300 V. In microfabrication, SiO_2_ and tetraethyl orthosilicate (TEOS) are typically used for thick dielectric layers. It is generally difficult to deposit SiO_2_ or TEOS at a thickness greater than 10 µm. It is also very difficult to pattern thick dielectric layers using UV-based lithography. Therefore, dielectric layers with a thickness of ~10 µm are widely used in surface ion traps.

### 3.2. Shape and Size of the Chip

**Figure 6:**
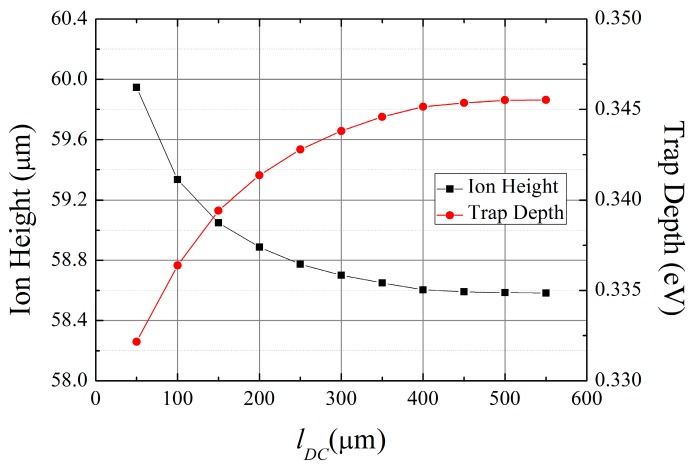
Simulation results for the trap depth and ion height as a function of the length of the DC electrodes. We fix *a* and *b* at 50 μm and 200 μm, respectively. The ion species assumed in the simulations is ^171^Yb^+^.

As shown in Equation (5), the beam radius increases with distance from the waist. Therefore, a smaller chip size is advantageous for preventing laser clipping at the chip edges. The main factor that restricts the chip miniaturization is the size and number of the DC electrodes. Wire bonding multiple electrodes to bonding pads requires sufficient lateral spaces on the electrode metal layer. To overcome this constraint, electrode routings using multi-layered metals and structures have been developed [46]. However, we do not consider this method in this study. As explained in Section 2.4, as dimension *b* increases, the simulation regions must be expanded to provide an outer ground plane of sufficient size. In the practical design of surface ion traps, the outer ground plane is separated into DC electrodes and the simulation regions can be determined by the lengths of the straight sections of the DC electrodes along the *x*-direction in Figure 2. To determine a sufficient length for these straight sections, we simulate the ion height and trap depth with different lengths of the DC electrodes. For these simulations, we fix the lengths *a* and *b* in Figure 2 at 50 μm and 200 μm, respectively. The simulation results shown in Figure 6 indicate that the variation rates of the trap depth and ion height decrease as the length of the DC electrodes is increased. Thus, the accuracy of the simulations and the length of the DC electrodes represent a trade-off problem. For example, the errors of the trap depth and ion height in the cases using 150-μm and 200-μm lengths for the DC electrodes are less than 1%. Additionally, the errors in the cases with 400-μm and 450-μm lengths of the DC electrodes are reduced to 0.2%.

### 3.3. Design of RF electrodes

Before designing the RF electrodes, we need to approximately calculate the constraint for the ion height. The beam diameter in Equation (5) is based on the “half-width at 1/*e*^2^ point” definition, which considers the intensity of the beam to be 1/*e*^2^ times the maximum intensity. However, to set the proportion of the clipped beam power at a specific value, we can use the definitions of beam intensity and power described below:

(8) $$I(x,y)=I_{0}\exp\left[\frac{-2(x^{2}+y^{2})}{w_{0}{}^{2}}\right],\;\;P=\iint I(x,y)dxdy$$

where *I*, *I_0_*, and *P* represent beam intensity, beam intensity at the center of the beam, and beam power, respectively. Note that the coordinates of *x* and *y* in Equation (8) represent Cartesian coordinates perpendicular to the beam path. Using these definitions, we can calculate the proportion of the clipped beam power as a function of ion height. Therefore, the constraint for the ion height can be calculated when we define a constraint for the proportion of the clipped beam power.

In the design procedure for the RF electrodes, the amplitude of the RF voltage is generally assumed to be the maximum value available for prohibiting electric breakdown, as described in Section 3.1. The design of the RF electrodes has three inputs, *a*, *b,* and Ω, and four outputs, trap depth, *q*-parameter, radial secular frequencies, and ion height. To simplify the design problem, we fix one output specification at a constant value. The output specification can be selected from among the trap depth, *q*-parameter, and radial secular frequency because these values can be easily tuned to a constant by adjusting the RF frequency. After the output specification is fixed, two inputs and three outputs remain. Then, we can investigate the characteristics of the remaining output specifications. The appropriate constraints for each specification are given considering the purpose of a particular chip design, and the electrode dimensions *a* and *b*, the two remaining inputs, are determined to satisfy the given constraints. In Section 4, we explain in detail the design method of the RF electrodes and present a case study.

### 3.4. Investigation of Effects of DC Voltages

After determining the lateral dimensions of the RF electrodes, we investigate the effects of the DC voltages at the ion position. This step is especially important because, generally, the output voltages of digital-to-analog converters are limited. As a first step, we must achieve an axial secular frequency greater than the constraint value. The constraint can be determined by considering the experimental goal and the available measurement systems. As the next step, we must determine the feasibility of tilting the principal axes near 45°. In order to tilt the principal axes of the ion’s secular motion, we apply DC voltages that are asymmetric along the *z*-axis to the outer DC electrodes. We can calculate the tilt angle by computing the Hessian matrix of the total trap potential and finding the eigenvalues. Finally, we must determine whether the electric potential from the center DC electrodes can compensate for the asymmetric electric potential of the outer DC electrodes. In other words, the total electric potential at the ion position generated by the DC electrodes must be zero.

As long as the lateral dimensions of the RF electrodes satisfy the conditions described above, the constraint dimensions of *a* and *b* can be modified to improve the specifications of the surface ion trap described in Section 2. Essentially, the electrode dimensions can be optimized by repetitively designing RF electrodes with different constraint dimensions and then investigating the effects of the DC voltages. If the designed electrode dimensions cannot satisfy the requirements, the design procedure of the RF electrodes must be repeated with a larger constraint dimension of *a* and a smaller dimension of *b* than in the previous design.

## 4. Case Study of the Design Methodology

**Figure 7:**
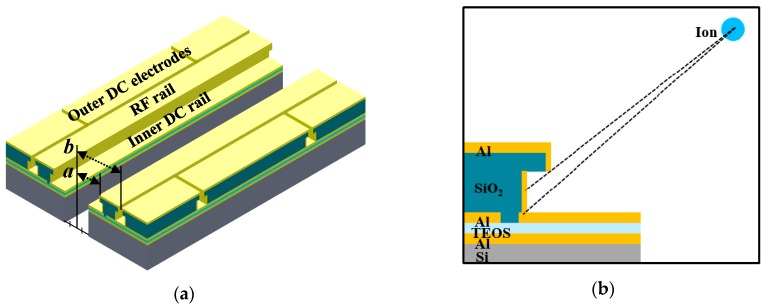
(**a**) Three-dimensional schematic of the surface ion-trap structures assumed in the case study and (**b**) a cross-section schematic of the surface ion trap under consideration. The structure is designed with metalized sidewalls to shield the trapped ions from the inevitable charges built up on the dielectric sidewalls.

In this section, we apply the design methodology described in the previous section to a particular trap-chip design. As a first step, we describe our basic assumptions. The surface ion-trap chip is designed to trap ^171^Yb^+^ ions. Accordingly, lasers with wavelengths of 369.5 nm, 399 nm, 638 nm, and 935 nm must be used for Doppler cooling and state detection (^2^S_1/2_ ↔ ^2^P_1/2_), photoionization, repumping from the ^2^F_7/2_ state, and repumping from the ^2^D_3/2_ state, respectively [47]. The chip is assumed to be mounted on the commercial chip package CPGA 10039 (Spectrum Semiconductor Materials, San Jose, CA, USA), with a mounting area of 1.2 cm × 1.2 cm and a total pin number of 100. Figure 7 shows a cross-section schematic of the surface ion trap under consideration. The structure is designed with metalized sidewalls to shield the trapped ions from the inevitable charges built up on the dielectric sidewalls. We place two inner DC rails inside the RF rails to tilt the principal axes of the ion’s secular motion. The inner DC rails are placed on the lower layer to reduce the background scattering of the laser. The silicon substrate between the inner DC rails is penetrated for loading neutral atoms from the backside of the trap surface. The dimension *a* is initially constrained by the condition *a* ≥ 40 μm, which provides sufficient space for laying the inner DC rails and the loading slot between the RF electrodes. The dimension *b* is constrained by the condition *b* ≤ 300 μm, which provides the distance between the ions and the outer electrodes. We design an asymmetric chip shape to reduce beam clipping from specific directions and fix the chip size along the assumed beam path at 2.26 mm. The number of outer DC electrodes, each with a width of 70 μm, is 24. These planar dimensions are based on a previous study [12]. More details for the design of the DC electrode can be found in [48].

**Figure 8:**
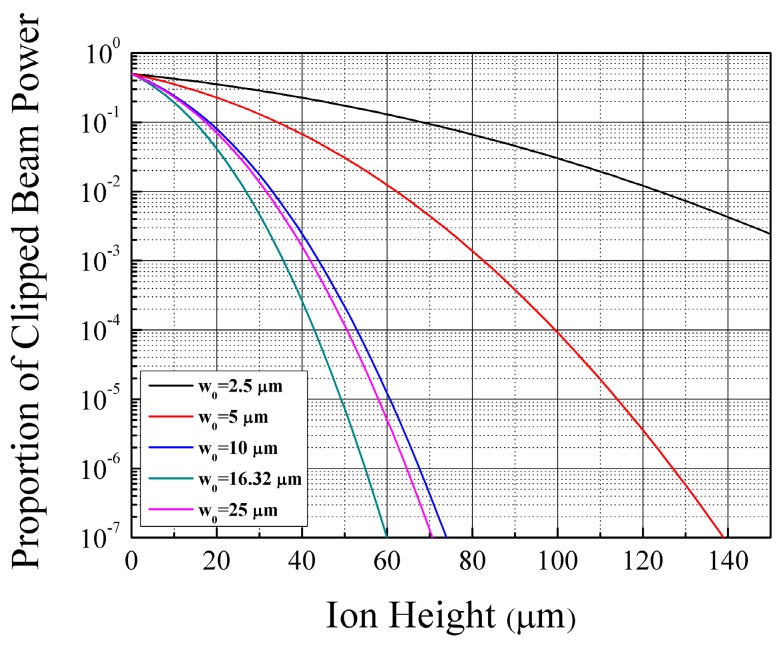
Proportion of the clipped beam power fraction as a function of ion height. For the calculation, we assume a laser with a wavelength of 369.5 nm, which is used in Doppler cooling and state detection of the ^171^Yb^+^ ion.

Figure 8 shows the proportion of the clipped beam power as a function of ion height. In the calcuation, we assume a laser with a wavelength of 369.5 nm, which is used in Doppler cooling and state detection of the ^171^Yb^+^ ion. As described in the Section 2.2.4, the disturbance to beam propagation by the chip body is minimized when the beam waist is equal to (*lλ/π*)^1/2^ , where *l* is the distance between the waist and the chip edge where beam clipping occurs. For example, assuming that the distance between the chip center and the edge is 2.26 mm and that the wavelength of the laser is 369.5 nm, the proportion of the clipped beam is minimized when the beam waist is 16.32 μm. For the 369.5-nm laser shown in Figure 8, the proportion of the clipped beam power is calculated to be 10^−6^ when the beam waist is 16.32 μm. Therefore, we can calculate the constraint for the ion height from Equation (8), and the calculated constraint value is 54.9 μm. We then use this value as a constraint when designing the RF electrodes.

**Figure 9:**
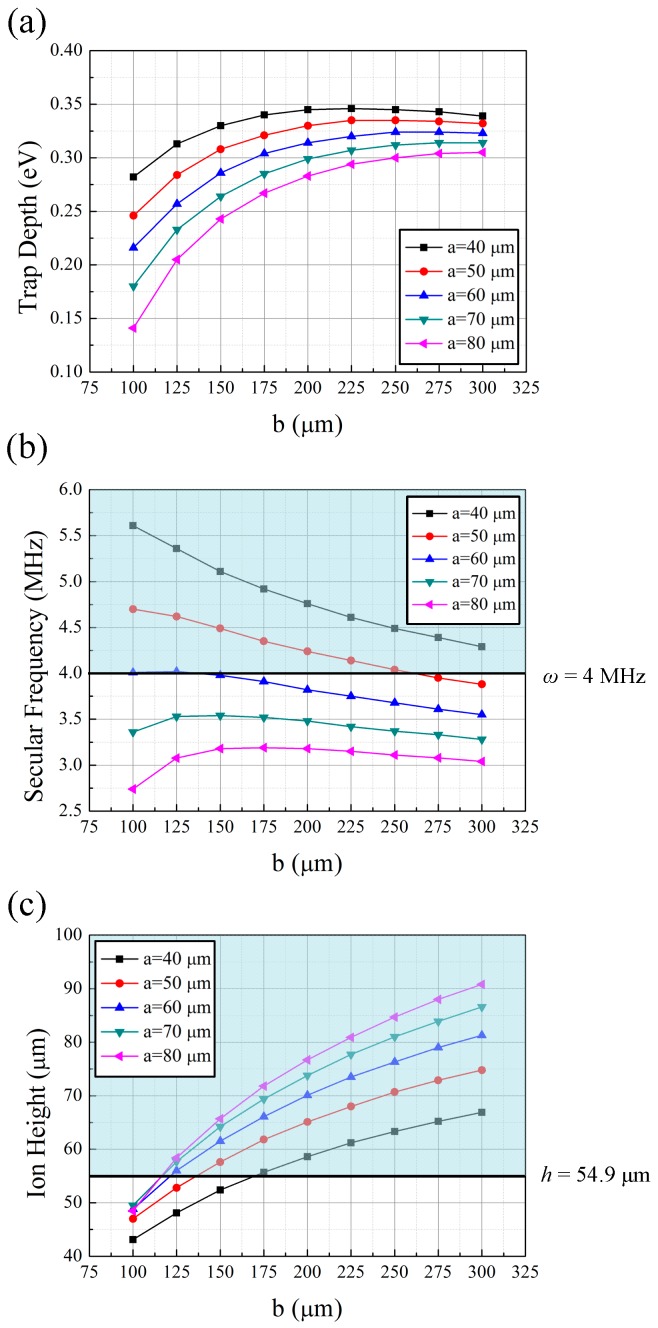
Simulation results for the case of trapping ^171^Yb^+^ ions, with the *q*-parameter fixed at 0.25, as functions of *a* and *b*. We tune the *q*-parameters to 0.25 in each simulation set by adjusting the RF frequency. The blue blocks represent the regions that satisfy the constraints considered. (**a**) trap depth; (**b**) radial secular frequency; (**c**) ion height.

Below, we describe an example design procedure for RF electrodes. As mentioned in Section 2.2.1, the goal of this design procedure is to maximize the trap depth. In this design scenario, we fix the *q*-parameter at a constant value of 0.25 and maximize the trap depth. The radial frequencies, ω*_s,x_* and ω*_s,y_*, which are the less stringent constraints, can be determined by the relation ω*_s,x_*/ω*_s,z_* > 0.73*N*^0.86^ and ω*_s,y_*/ω*_s,z_* > 0.73*N*^0.86^, where *N* represents the number of ions in the ion string [49]. If arbitrary conditions *N* = 14 and ω*_s,z_* = 551 kHz are considered as an example case, the secular frequency must be higher than 4 MHz. Another less stringent constraint, the ion height, is restricted to be greater than 54.9 µm, as explained in the previous paragraph. Figure 9 shows the simulation results with the *q*-parameter fixed at 0.25. We tune the *q*-parameter to 0.25 in each simulation by adjusting the RF frequency. As shown in Figure 9a,b, small *a* values provide high trap depths and secular frequencies. Based on these simulation results, we select a value of *a* = 40 µm. At this value, the secular frequency is larger than the design constraint value, 4 MHz, regardless of the *b* value. With regard to ion height, *b* must be smaller than 164 µm (Figure 9c). Thus, we select *b* = 164 µm to maximize the trap depth while satisfying the constraint on ion height. In conclusion, the expected values of the final specifications are a trap depth of 0.311 eV at an RF frequency of 56.48 MHz, a secular frequency of 4.80 MHz, and an ion height of 54.9 µm, when *a* = 40 µm and *b* = 164 µm are chosen as the dimensions of the RF electrodes.

**Figure 10:**
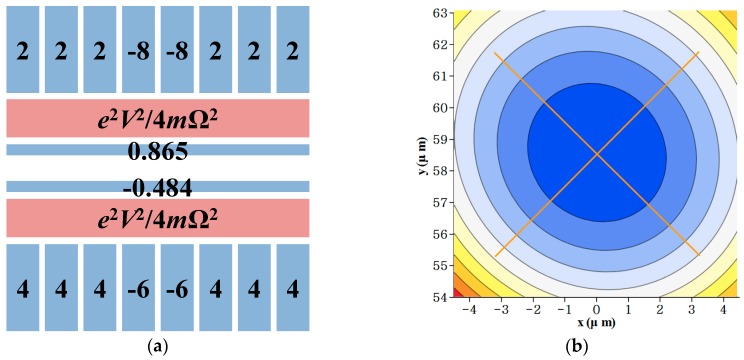
Investigation of the effects of the DC voltages at the ion position. (**a**) applied DC voltage set; (**b**) axes tilt result of 44.7°. The tilt angle is achieved by computing the Hessian matrix of the total trap potential and finding the eigenvalues.

Figure 10 shows a DC voltage set for trapping ions and the result for the tilted principal axes. When we use the voltage set in Figure 10a, the total electric potential at the ion position is zero. In addition, we obtain an axial secular frequency of 551 kHz and a principal axes tilt of 44.7°. Note that the absolute values of the voltage amplitudes are under 10 V (the peak limited by digital-to-analog converters in our case). If the simulation results including the DC voltages do not satisfy the necessary conditions of each experimental setup, the constraint dimensions for *a* and *b* must be adjusted and the design of the RF electrodes must be repeated.

## 5. Conclusions

In this paper, we presented a design methodology and a case study for designing the electrodes of surface ion traps. We briefly presented the theory behind surface ion traps and explained their specifications. We showed the accuracy and necessity of using the BEM tool for designing an ion trap by comparing analytic solutions with our simulation results. The design methodology presented in this paper comprises the following four steps: setting the basic assumptions, determining the shape and size of the chip, designing the dimensions of the RF electrodes, and investigating the effects of the DC voltages at the ion position. Based on the basic assumptions and chip size, the lateral dimensions of the RF electrodes can be designed. Then, we investigated the validity of the RF and DC electrode designs by inspecting the axial secular frequency, the principal axes tilt, and the total electric potential at the ion position. The design process can be iteratively carried to optimize the required specifications and constraints.

We also explained the application of our design methodology by using a case study. The length of the DC electrodes was set at 400 μm. To set the proportion of the clipped beam power to under 10^−6^, we calculated the required minimum ion height to be 54.9 μm for the ^171^Yb^+^ ion. The beam waist was assumed to be 16.32 μm to minimize the beam radius at the chip edge. We carried out the design procedure with the following conditions: a *q*-parameter of 0.25, a secular frequency value greater than 4 MHz, and an ion height of more than 54.9 μm. Using BEM simulations, an electrode geometry with *a* = 40 μm and *b* = 164 μm resulted in a trap depth of 0.311 eV at an RF frequency of 56.48 MHz, a secular frequency of 4.80 MHz, and an ion height of 54.9 μm. We achieved a principal axes tilt of 44.7° with DC voltages of less than 10 V. For different experimental objectives, an optimized design for each case scenario can be obtained using the design methodology outlined in this paper. The developed design methodology can be applied to surface ion traps with more complex structures and those for trapping multiple species.

## Figures and Tables

**Table 1:** Electrode dimensions and specifications of surface ion traps.

Ref No.	*a* (µm)	*b* (µm)	V_RF_ (V)	Ω_RF_/2π (MHz)	Ion Species	Trap Depth (meV)	*q*-Parameter	Radial Secular Frequency (MHz)	Ion Height (µm)
[12]	77	137	50–140	33	^40^Ca^+^	-	0.25–0.34 *	3–4	84
[21]	100	247	223	25.8	Ca^+^	188	0.43	4.02	150
[24]	asymmetric		100	60	^40^Ca^+^	-	0.16–0.19 *	3.5–4	63
[25]	asymmetric		51	90.7	^24^Mg^+^	-	0.25 *	8	40
[27]	44	84	91	58.55	^40^Ca^+^	-	0.05–0.12 *	1–2.5	60
[32]	75	95	155	40.6	^88^Sr^+^	25	0.15 *, 0.12 *	2.1, 1.7	79
[33]	45	136	72	38.7	^43^Ca^+^	59	0.3	4	75
[34]	asymmetric		140	20.6	^40^Ca^+^	75	-	-	230

* These numbers were estimated using $q\approx 2\sqrt{2}\frac{\omega_{s}}{\Omega_{RF}}$.

## Abbreviations

The following abbreviations are used in this manuscript:

AC	Alternating current
BEM	Boundary element method
DC	Direct current
FEM	Finite element method
RF	Radio frequency
SEM	Scanning electron micrograph
TEOS	Tetraethyl orthosilicate
UV	Ultra violet
